# Nitric oxide promotes cysteine N-degron proteolysis through control of oxygen availability

**DOI:** 10.1073/pnas.2501796122

**Published:** 2025-08-19

**Authors:** Haeun Kim, Ya-Min Tian, Peter J. Ratcliffe, Thomas P. Keeley

**Affiliations:** ^a^Target Discovery Institute, Nuffield Department of Medicine, University of Oxford, Oxford OX3 7FZ, United Kingdom; ^b^Ludwig Institute for Cancer Research, Nuffield Department of Medicine, University of Oxford, Oxford OX3 7DQ, United Kingdom; ^c^The Francis Crick Institute, London NW1 1AT, United Kingdom; ^d^Department of Physiology, Anatomy and Genetics, University of Oxford, Oxford OX1 3PT, United Kingdom

**Keywords:** hypoxia, nitric oxide, proteolysis, N-degron, oxygen

## Abstract

Selected proteins containing an N-terminal cysteine (Nt-Cys) are subjected to rapid, O_2_-dependent proteolysis via the Cys/Arg-branch of the N-degron pathway. Cysteine dioxygenation is catalyzed in mammalian cells by 2-aminoethanethiol dioxygenase (ADO), an enzyme that manifests extreme O_2_ sensitivity. The canonical substrates of this pathway in mammalia are the regulators of G-protein signaling 4, 5, and 16, as well as interleukin-32. In addition to operating as an O_2_-sensing mechanism, this pathway has previously been described as a sensor of nitric oxide (NO), with robust effects on substrate stability upon modulation of NO bioavailability being widely demonstrated. Despite this, no mechanism to describe the action of NO on the Cys/Arg N-degron pathway has yet been substantiated. We demonstrate that NO can regulate the stability of Cys N-degron substrates indirectly via the regulation of ADO cosubstrate availability. Through competitive, O_2_-dependent inhibition of cytochrome C oxidase, NO can substantially modify cellular O_2_ consumption rate and, in doing so, alter the availability of O_2_ for Nt-Cys dioxygenation. We show that this increase in O_2_ availability in response to NO exposure is sufficient to alter both dynamic and steady-state ADO substrate levels. It is likely that this mechanism operates to couple O_2_ supply and mitochondrial respiration with responses to G-protein-coupled receptor stimulation.

## Significance

Oxygen homeostasis in complex animals requires multiple sensory mechanisms to integrate cell-autonomous actions with tissue- and system-wide feedback circuits. 2-aminoethanethiol dioxygenase (ADO)-catalyzed N-degron proteolysis is rapid, highly O_2_ sensitive, and capable of dynamically regulating cellular function in response to changing O_2_ levels. A role for NO in this pathway has long been appreciated, but no clear mechanism for this action has yet been described. Here, we demonstrate that NO has a potent effect on intracellular O_2_ availability through inhibition of mitochondrial O_2_ consumption and, by doing so, strongly influences the rates of reactions catalyzed by highly O_2_-sensitive enzymes such as ADO, with wide-reaching implications for cell and tissue O_2_ homeostasis.

The biochemistry and physiology of nitric oxide (NO) and oxygen (O_2_) are intricately linked. Physiological production of NO in mammalian cells requires O_2_ as a substrate (1), and these gases react rapidly with each other to form highly reactive nitrogen species when either or both are in excess (2–4). Given this complex biochemistry, it is not surprising that interactions with NO are commonly described in most known mechanisms of O_2_ sensing, including direct inhibition of hypoxia-inducible factor (HIF) prolyl hydroxylase domain (PHD) enzymes (5–7), competitive antagonism at cytochrome C oxidase (8), and hypoxic vasodilation through *S*-nitrosohemoglobin (9).

A role for NO in the proteolytic regulation of proteins bearing an N-terminal cysteine (Nt-Cys) residue has also long been appreciated. Nt-Cys dioxygenation is the second step in the regulated degradation of proteins via the Cys branch of the N-degron pathway. Following methionine cleavage, susceptible Nt-Cys are rapidly dioxygenated in the presence of O_2_ to cysteine sulfinic acid (-SO_2_H), a process catalyzed in plants by plant cysteine oxidases (PCO) (10–13) and in animals by 2-aminoethanethiol dioxygenase (ADO) (14). This newly formed motif is recognized and arginylated by arginyl t-RNA transferase 1 (ATE1), producing a canonical arginine N-degron that targets the protein for ubiquitination by UBR1/2 in animals and PROTEOLYSIS 6 in plants, and subsequent proteasomal degradation (15–17). In seminal work (18), it was shown that exogenous addition of NO promoted the degradation of regulators of G-protein signaling (RGS)4, 5, and 16 via a modification of the N-terminal residue which rendered the protein susceptible to arginylation by ATE1 in vitro. Similarly, chemical chelation of NO using 2-4-carboxyphenyl-4,4,5,5-tetramethylimidazoline-1-oxyl-3-oxide (cPTIO) resulted in an increase in detectable RGS4 in cells in which NO was produced endogenously (18). Subsequent work placed these biochemical observations in a pathophysiological context, suggesting that NO-mediated stimulation of RGS4 proteolysis regulates angiogen-induced cardiomyocyte hypertrophy (19). In plants, NO was shown to modify the stability of group VII ethylene response factors (ERFVII) to regulate germination (20) and abiotic stress (21). Moreover, rapid hypoxia-mediated upregulation of the endogenous NO scavenger phytoglobin1 was shown to regulate Cys N-degron substrate stability for enhanced tolerance to submergence (22). We previously demonstrated that NO does not affect the stability of RGS4 or 5 in mammalian cells in which ADO has been genetically silenced (14), in contrast with the concept of spontaneous Nt-Cys oxidation as a mechanism by which NO altered the stability of RGS4/5.

Here, we have systematically tested the effect of NO on each step in the mammalian Cys N-degron pathway, demonstrating that Nt-Cys dioxygenation can occur under conditions in which significant levels of NO are unlikely to be present, and confirming that NO does not influence N-degron processing downstream of ADO. Instead, we demonstrate that NO reduces cellular O_2_ consumption through competitive antagonism at cytochrome C oxidase and, in doing so, increases cellular O_2_ availability to such an extent that it manifests as changes in ADO substrate stability.

## Results

### ADO-Dependent Modulation of RGS4 Stability by NO.

**Fig. 1.**
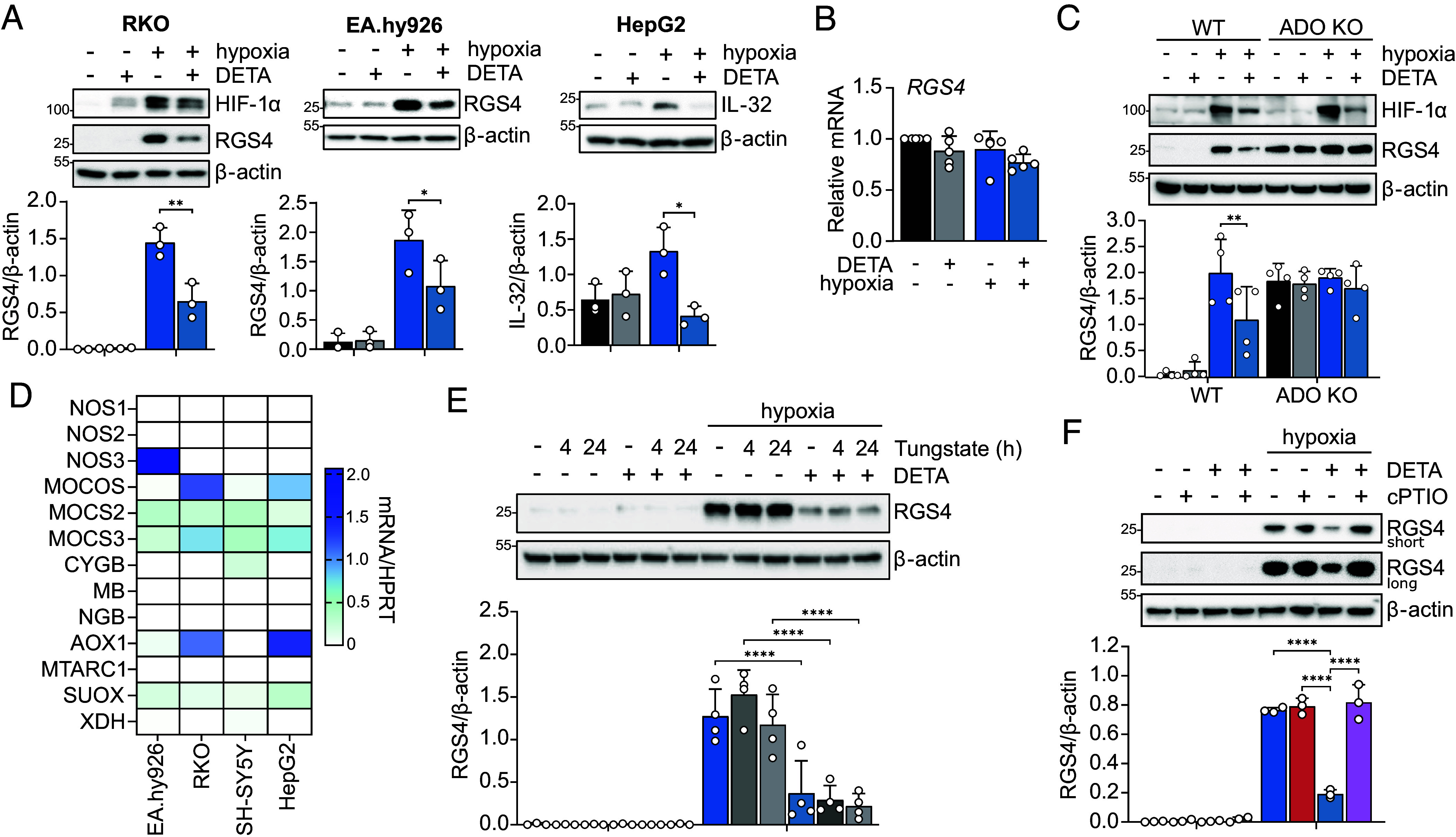
NO as a modulator of the Cys N-degron pathway. (*A*) Representative immunoblots of the indicated proteins from RKO, EA.hy926, and HepG2 cells treated with DETA-NONOate (100 μM) for 4 h under normoxic or hypoxic (1% O_2_) conditions. Densitometric analysis from three independent experiments is provided below as the mean ± SD. *P < 0.05, **P < 0.01, two-way ANOVA with Holm–Šídák’s multiple comparisons post hoc test. (*B*) RT-qPCR analysis of RGS4 mRNA levels in RKO cells treated with DETA for 4 h under normoxic or hypoxic (1% O_2_) conditions. Data plotted are the mean ± SD from five independent experiments. (*C*) Representative immunoblots for HIF-1α and RGS4 protein in wild-type RKO cells or RKO cells that have undergone genetic inactivation of ADO, treated as above. Densitometric analysis from four independent experiments is provided below as the mean ± SD. **P < 0.01, two-way ANOVA with Holm–Šídák’s multiple comparisons post hoc test. (*D*) RT-qPCR analysis of transcripts encoding the three nitric oxide synthase (NOS) isoforms, as well as those important for the reduction of nitrite to NO (MOCOS–molybdenum cofactor sulfurase, MOCS2/3–molybdopterin synthase catalytic subunit 2/3, CYGB–cytoglobin, MB–myoglobin, NGB–neuroglobin, AOX1–aldehyde oxidase 1, MTARC1–mitochondrial amidoxime-reducing component 1, SUOX–sulfite oxidase, XDH–xanthine dehydrogenase) in EA.hy926, RKO, SH-SY5Y, and HepG2 cells. Data are visualized as the mean from three independent experiments. (*E*) RGS4 protein levels in RKO cells treated with tungstate (0.5 mM) for 20 h prior to, or alongside (4 h), exposure to hypoxia in the presence or absence of DETA. Representative immunoblots are shown above, and densitometric analysis from 3 independent experiments is provided below as the mean ± SD. ****P < 0.0001, two-way ANOVA with Holm–Šídák’s multiple comparisons post hoc test. (*F*) RGS4 protein levels in RKO cells treated with DETA or the NO-chelator cPTIO (200 μM), alone or in combination under either normoxia or hypoxia, for 4 h. Representative immunoblots are shown above, including a higher exposure immunoblot to verify the absence of detectable RGS4 in normoxic cells, and densitometric analysis from three independent experiments is provided below as the mean ± SD. ****P < 0.0001 vs. normoxic control, two-way ANOVA with Holm–Šídák’s multiple comparisons post hoc test.

As a first step, we set out to characterize the conditions under which NO influenced the levels of ADO substrates. In RKO cells, the accumulation of RGS4 protein during a short incubation under hypoxia (4 h, 1% O_2_) was significantly reduced if cells were concurrently treated with a slow-release NO donor ((Z)-1-[N-(2-aminoethyl)-N-(2-ammonioethyl)amino]diazen-1-ium-1,2-diolate (DETA-NONOate), 100 μM, Fig. 1*A*). This concentration is known to evoke similar levels of cyclic-guanosine monophosphate production in human umbilical vein endothelial cells to that induced by exposure to physiological fluid shear stress or Gα_q_-coupled agonists, suggesting a release of NO within the physiological range (23). Such actions were also observed in a different cell line (EA.hy926) and on a different ADO substrate (IL-32 in HepG2, Fig. 1*A*). Importantly, while NO is known to stabilize HIF-1α under normoxia (6, 24), we observed no such effect on RGS4 or IL-32, suggesting that NO might have both common and distinct interactions with PHD- and ADO-catalyzed O_2_-sensing pathways. Reductions in RGS4 protein following DETA treatment were not due to alterations in mRNA levels (Fig. 1*B*). Moreover, genetic inactivation of ADO results in constitutive stabilization of RGS4 (14), and exposure of ADO-deficient cells to NO had no further effect on RGS4 protein levels under either normoxia or hypoxia (Fig. 1*C*).

It was originally proposed that endogenous NO production is necessary for Cys N-degron proteolysis (18). While many of the cell lines used here and in previous work (14, 25) expressed minimal levels of mRNA transcript encoding for nitric oxide synthase isoforms (NOS1/2/3, Fig. 1*D*), all expressed one or more transcripts encoding for proteins required to generate NO via nitrite reduction. This included all components required to synthesize the molybdopterin cofactor (MOCOS–molybdenum cofactor sulfurase, MOCS2/3–molybdopterin synthase catalytic subunit 2/3), as well as enzymes possessing nitrite reductase activity (AOX1–aldehyde oxidase 1, SUOX–sulfite oxidase). Notably, this pathway has recently been shown to be particularly prominent under conditions of O_2_ depletion (26, 27). To test whether NO generated through nitrite reduction was contributing to Cys N-degron proteolysis, we incubated RKO cells with tungstate, which inhibits nitrite reductases by replacing their molybdenum cofactor (27). Cells were exposed to tungstate (0.5 mM) for either 20 h prior to, or contemporaneously with, hypoxic exposure in the presence or absence of DETA, and RGS4 protein accumulation was assessed. Pretreatment was included to ensure sufficient time for molybdenum cofactor substitution. As shown in Fig. 1*E*, neither acute nor sustained exposure to tungstate had any effect on RGS4 stability under normoxic or hypoxic conditions. To address the possibility that NO generated from an unknown source contributes to this reaction, RKO cells were treated under normoxic or hypoxic conditions with the NO scavenger cPTIO. The presence of cPTIO had no effect on RGS4 levels under either normoxia or hypoxia, but did abolish the effect of DETA treatment (Fig. 1*F*). Taken together, these findings indicate that NO may exert a potent effect on ADO substrate stability under hypoxia and suggest it may not be necessary for enzyme-catalyzed Nt-Cys proteolysis.

### Downstream N-Degron Processing Is Unaffected by NO.

**Fig. 2.**
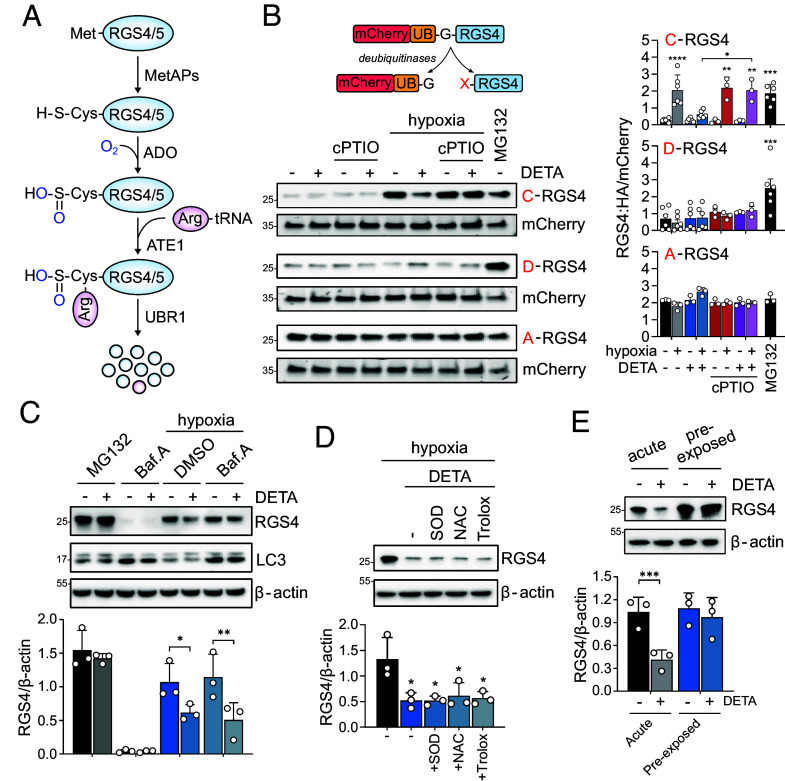
NO does not alter Cys N-degron processing downstream of ADO. (*A*) A schematic illustrating the Cys-branch of the N-degron pathway; MetAPs–methionine aminopeptidases, ATE1–arginyl tRNA transferase, UBR1–UBR box recognin 1. (*B*, *Top*) the ubiquitin protein reference technique, in which the action of endogenous deubiquitinases cleave the UBQ moiety to separate the stable mCherry reference protein from either WT RGS4 (C-RGS4) or a mutant in which the N-terminal residue was mutated to an aspartate (D-RGS4) or alanine (A-RGS4). (*Lower*) Representative immunoblots from HEK293T cells transfected with either C-, D-, or A-RGS4 and subjected to 4 h of hypoxia in the presence or absence of DETA, cPTIO, or both. Normoxic cells were also treated with MG132 (12.5 μM) as a positive control. Densitometric analysis from 3 to 6 independent experiments is provided on the right as the mean ± SD. *P < 0.05, **P < 0.01, ***P < 0.001, ****P < 0.0001 vs. normoxic control, two-way ANOVA with Holm–Šídák’s multiple comparisons post hoc test. (*C*) RGS4 protein levels in RKO cells treated with either MG132 or Bafilomycin A (Baf.A, 200 nM) under normoxic or hypoxic conditions for 4 h, and in the presence or absence of DETA. Representative immunoblots are shown above, and the analysis of three independent experiments provided below as the mean ± SD. *P < 0.05, **P < 0.01, two-way ANOVA with Holm–Šídák’s multiple comparisons post hoc test. Expression of LC3 was used as a positive control to indicate inhibition of lysosomal degradation by Baf.A. (*D*) RGS4 protein levels in RKO cells treated with DETA under hypoxic conditions for 4 h in the presence or absence of the antioxidants PEGylated superoxide dismutase (SOD, 100 U mL^−1^), N-acetyl cysteine (NAC, 500 μM), or trolox (100 μM). Representative immunoblots are shown above, and the analysis of three independent experiments provided below as the mean ± SD. *P < 0.05, one-way ANOVA with Holm–Šídák’s multiple comparisons post hoc test. (*E*) Cells were kept at either normoxia (acute) or hypoxia (pre-exposed) for 16 h before being treated with DETA and exposed to hypoxia for a further 4 h. Representative immunoblots are shown above, and the analysis of three independent experiments provided below as the mean ± SD. ***P < 0.001, two-way ANOVA with Holm–Šídák’s multiple comparisons post hoc test.

Since NO appeared likely not to be an obligate participant in Nt-Cys proteolysis, we next sought to systematically test at which point in the Cys N-degron pathway (Fig. 2*A*) it is operating. Removal of the initiating methionine residue by methionine aminopeptidases (MetAPs) is required to expose the Nt-Cys residue. We therefore first assessed whether NO influenced this reaction by assaying for an impact on the stability of a MetAP-independent version of RGS4 constructed using the ubiquitin protein reference technique (28). As shown in Fig. 2*B*, the UBQ-C-RGS4:HA reporter protein was stabilized following exposure to hypoxia and showed similar sensitivity to NO to that of its MetAP-dependent endogenous counterpart (Fig. 1), indicating that NO must act downstream of methionine excision. Since genetic inactivation of ADO precludes all subsequent processing of the Nt-Cys degron motif (14), it remained unclear whether NO influences ADO activity or that of the downstream enzymes ATE1 or UBR1/2. To test this, we generated another UBQ-RGS4:HA reporter protein in which the neo-Nt-Cys was mutated to an aspartate (D-RGS4:HA). This reporter protein should act as a substrate for ATE1 and downstream N-degron-dependent proteolysis, but critically should not be dependent on the action of ADO. As shown in Fig. 2*B*, the stability of D-RGS4:HA was unaffected by hypoxia or NO alone, or in combination, but was robustly stabilized by MG132 treatment. To rule out NO-dependent regulation of RGS4 that specifically occurs downstream of cysteine dioxygenation but upstream of arginylation, we then assessed the stability of an alanine RGS4:HA mutant (A-RGS4:HA), the regulation of which would be decoupled from the Arg/Cys N-degron pathway (Fig. 2*B*). As expected, this construct was more stable than C- or D-RGS4:HA equivalents and was unaffected by hypoxia, NO, or MG132. Taken together, these experiments indicate that the action of NO on Nt-Cys pathway substrate stability is at the level of ADO activity, and unlikely to involve an influence on iMet cleavage, or on downstream proteolytic processing via the Arg N-degron pathway.

A noncanonical lysosomal degradation pathway has been reported to target Nt-Cys-containing proteins (29). To address whether NO might promote lysosomal degradation of RGS4 specifically under hypoxia, we treated cells with either the proteasomal inhibitor MG132 or the lysosomal inhibitor bafilomycin A in the presence or absence of DETA, and under normoxic or hypoxic conditions (Fig. 2*C*). Consistent with an action of NO upstream of proteolysis, treatment with MG132 resulted in stabilization of RGS4 protein which was unaffected by cotreatment with DETA. While bafilomycin treatment was sufficient to inhibit autophagy, as evidenced by an increase in detectable LC3-II accumulation (Fig. 2*C*), it had no detectable effect on RGS4 levels either under normoxic or hypoxic conditions, consistent with sole regulation via N-degron-mediated proteolysis.

To further confirm that NO does not react with either O_2_ or any free radical derivatives to spontaneously oxidize the Nt-Cys of RGS4, we tested whether cotreatment with various scavengers of reactive oxygen species could reverse the effect of NO (Fig. 2*D*). PEGylated superoxide dismutase is a potent antioxidant enzyme used widely to scavenge O_2_^●^ radicals (30), whereas N-acetyl-L-cysteine and trolox (a vitamin E analogue) are more general redox scavengers. Cotreatment with these compounds had no effect on RGS4 stability under hypoxia in the presence of NO. Moreover, the addition of NO had little effect on RGS4 stability in cells pre-exposed to hypoxia (Fig. 2*E*), indicating it is not sufficient to stimulate oxidation and subsequent degradation in the absence of O_2_. These data, in conjunction with the observation that NO was ineffective in the absence of ADO [Fig. 1*C* and (14)], suggest that the Cys-SO_2_H N-degron motif is unlikely to be generated through a spontaneous redox reaction between physiological levels of NO and O_2_.

### Modulation of Intracellular O_2_ Levels by NO.

**Fig. 3.**
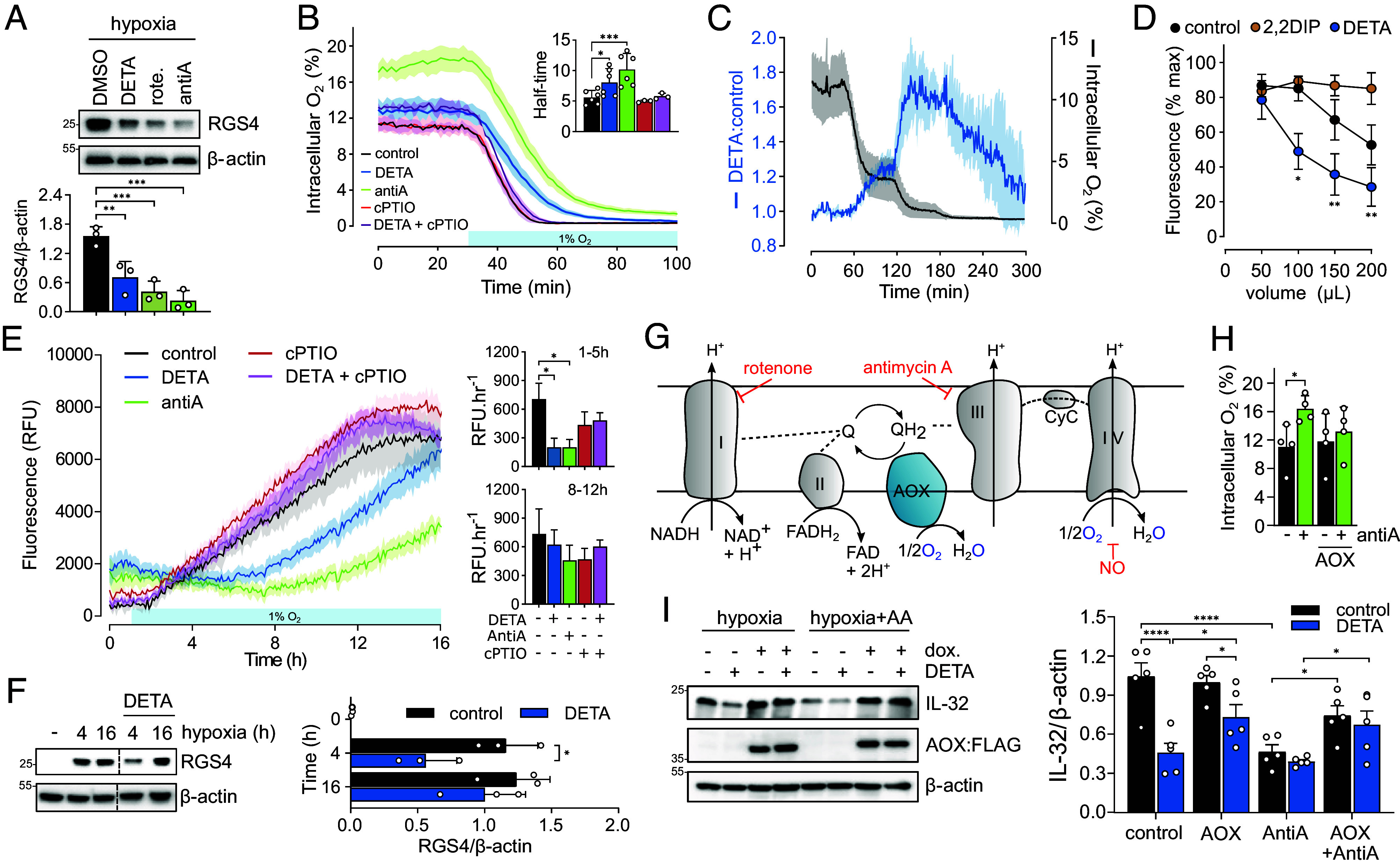
Modulation of mitochondrial O_2_ consumption alters RGS4 accumulation under hypoxia. (*A*) RGS4 protein levels in RKO cells exposed to 4 h hypoxia (1% O_2_) in the presence of either dimethyl sulfoxide (DMSO) vehicle (0.1%), DETA (100 μM), rotenone (Rot. 1 μM), or antimycin A (Anti.A, 5 μM). Representative immunoblots are shown above, and the analysis of three independent experiments provided below as the mean ± SD. **P < 0.01, ***P < 0.001, one-way ANOVA with Holm–Šídák’s multiple comparisons post hoc test. (*B*) Intracellular O_2_ levels ([O_2_]_i_) were monitored continuously using MitoXpress INTRA-based time-resolved phosphorescence in RKO cells treated with either DETA, cPTIO, DETA+cPTIO, or antimycin A and then exposed to 1% O_2_ when indicated. Kinetic data were fitted using regression analysis to obtain a deoxygenation time constant (half-time, *Inset*). Representative examples are shown in the line graph as the mean ± SD from 3 to 4 replicate wells, while the histogram *Inset* is the mean ± SD from 3 to 6 independent experiments. *P < 0.05, ***P < 0.001 one-way ANOVA. (*C*) [O_2_]_i_ in control or DETA-treated RKO cells subjected to step-wise reductions in ambient O_2_ levels. The relationship between [O_2_]_i_ in DETA- vs. control-treated cells is plotted as a ratio on the left *y*-axis, and [O_2_]_i_ in control cells plotted on the right *y*-axis. Data plotted are the mean ± SD from three independent experiments. (*D*) GFP fluorescence from RKO cells stably expressing RGS4_1-11_GFP following exposure to hypoxia (4 h, 1% O_2_) with 50 to 200 μL medium per well in the presence or absence of DETA. Cells were also exposed to 2,2DIP (100 μM) for comparison with an O_2_-independent stimulus. Data plotted are the mean ± SD from three independent experiments, *P < 0.05, **P < 0.01, vs. equivalent control, two-way ANOVA with Holm–Šídák’s multiple comparisons post hoc test. (*E*) GFP fluorescence was monitored in real-time following exposure to 1% O_2_ in cells treated with DETA, cPTIO, DETA+cPTIO, or Antimycin A. Rate constants were fitted to kinetic data between 1 to 5 h and 8 to 12 h, and plotted in the *Inset*. Data shown in the line graph are the mean ± SD from three replicate wells of a representative experiment, whereas the histogram *Inset* is the mean ± SD from 3 to 6 independent experiments. *P < 0.05 vs. equivalent control, two-way ANOVA with Holm–Šídák’s multiple comparisons post hoc test. (*F*) RGS4 protein levels in RKO cells exposed to hypoxia for 4 or 16 h in the presence or absence of DETA. Representative immunoblots are shown on the left, and the analysis of three independent experiments provided on the right as the mean ± SD. *P < 0.05, one-way ANOVA with Holm–Šídák’s multiple comparisons post hoc test. (*G*) A schematic of the electron transport chain illustrating the sites of action of the inhibitors rotenone, antimycin A, and NO, as well as the location and function of AOX. (*H*) Intracellular O_2_ levels in RKO cells expressing inducible FLAG-tagged AOX and treated with 1 μM antimycin A for 1 h. Data represent the mean ± SD from four independent experiments, *P < 0.05, two-way ANOVA with Holm–Šídák’s multiple comparisons post hoc test. (*I*) IL-32 protein levels in RKO cells expressing inducible FLAG-tagged AOX and exposed to 3% O_2_ for 4 h in the presence or absence of 100 μM DETA and/or 1 μM antimycin A. A representative immunoblot is shown on the left, and the analysis of five independent experiments is provided on the right as the mean ± SD, *P < 0.05, ****P < 0.0001, two-way ANOVA with Holm–Šídák’s multiple comparisons post hoc test.

The data presented so far suggest that NO regulates RGS4 stability through a regulatory action on ADO activity within the cellular environment. NO is known to compete with O_2_ for binding at the heme center of cytochrome C oxidase (8), and inhibition of mitochondrial O_2_ consumption has been proposed as a mechanism underpinning the effects of NO on HIF-1α stability under physiological conditions (31). If the same were true of the ADO system, other inhibitors of mitochondrial O_2_ consumption would be predicted to have a similar effect on RGS4 accumulation. Indeed, similar effects of modulators of mitochondrial respiration on Cys N-degron substrate stability have been more recently demonstrated on the plant N-degron pathway (32). To test this, we exposed RKO cells to hypoxia in the presence or absence of NO, rotenone (1 μM, a complex I inhibitor), or antimycin A (5 μM, a complex III inhibitor) for 4 h. As shown in Fig. 3*A*, both respiratory chain inhibitors were even more potent at reducing RGS4 accumulation than NO. To better correlate the action of NO and mitochondrial inhibitors on RGS4 stability with changes in O_2_ availability, we directly measured intracellular O_2_ levels ([O_2_]_i_) in cells during exposure to hypoxia using O_2_-sensitive phosphorescent nanoparticles (30, 33) (Fig. 3*B*). While treatment of cells with antimycin A resulted in a higher [O_2_]_i_ under both normoxic and hypoxic conditions, NO treatment had little impact on [O_2_]_i_ under normoxic conditions. However, treatment with either NO or antimycin A did significantly delay the rate of deoxygenation when cells were subsequently exposed to hypoxia (Fig. 3 *B*, *Inset*). Consistent with a lack of effect on RGS4 accumulation (Fig. 1*F*), cPTIO treatment had no effect on intracellular O_2_ levels in RKO cells, but did attenuate the effect of DETA (Fig. 3*B*). To better understand the relationship between NO, O_2_ availability, and O_2_ consumption, RKO cells were treated with DETA and subjected to step-wise reductions in ambient O_2_ level. As shown in Fig. 3*C*, the influence of NO on intracellular O_2_ availability (represented as the ratio of [O_2_]_i_ in DETA-treated cells to that of control cells) was highly dependent on [O_2_]_i_, with the greatest effect observed within the physiological range (1 to 5% O_2_).

When temperature and monolayer O_2_ consumption are consistent, O_2_ diffusion to the cell monolayer is principally dependent on diffusion distance, which is proportional to the volume of medium within a cylindrical culture well (34). Reducing this volume can therefore compensate for reductions in O_2_ consumption, and hence, it may attenuate the effect of NO on ADO substrate stability. Decreasing medium volume (from 200 to 50 μL) increased the amount of an RGS4_1-11_GFP reporter protein (35) in RKO cells following exposure to 4 h hypoxia (Fig. 3*D*). When cells were treated with NO, the effect on hypoxia-induced RGS4_1-11_GFP accumulation reduced proportionally with medium volume, and was no longer evident with ≤50 μL medium (Fig. 3*D*). In contrast, the action of an Fe^2+^ chelator (2,2DIP) on reporter accumulation was insensitive to changes in medium volume.

While slowing the rate of deoxygenation upon exposure to hypoxia may impact short-term RGS4 accumulation, cells eventually reach a sufficient level of hypoxia to render ADO largely inactive. Hence, substrate would eventually reach similar a steady-state level if exposed to hypoxia for a sufficiently long enough period. This hypothesis was tested using the RGS4_1-11_GFP reporter protein to monitor ADO substrate accumulation in real-time during hypoxic exposure. As shown in Fig. 3*E*, RGS4_1-11_GFP began accumulating within 1 h of exposure to hypoxia and plateaued 16 h thereafter, approximating the kinetics of endogenous substrate accumulation (25). In contrast, the rate of reporter accumulation was significantly lower within the initial hypoxia period (1 to 5 h) in cells treated with DETA or antimycin A, but it became comparable following prolonged hypoxia (8 to 12 h, Fig. 3*E*). cPTIO alone had no effect on reporter accumulation, but did reverse the effect of exogenously applied NO. Similar effects of NO were observed when endogenous RGS4 levels were analyzed by immunoblotting after 4 or 16 h of hypoxia (Fig. 3*F*).

The mitochondria of bacteria, plants, and several species of invertebrates possess a mechanism to prevent build-up of reduced quinone within the electron transport chain (ETC), as would occur when complex IV is inhibited. This involves an alternative oxidase (AOX) found proximal to complex III that can directly reduce O_2_ to H_2_O using QH_2_, thereby maintaining a flowing ETC and proton gradient albeit at reduced capacity (Fig. 3*G*). Importantly, AOX is reported to be insensitive to both cyanide (36) and NO (37) and can maintain a modest O_2_ consumption when COXIV is inhibited in mammalian cells (36, 38). We therefore reasoned that supplementing cells with AOX might alleviate the effects of NO on mitochondrial respiration and in doing so would lessen its impact on RGS4 accumulation under hypoxia. FLAG-tagged AOX from *Candida albicans* (UniProt–O93853) (39) was expressed in RKO cells under a doxycycline inducible promoter, and its function was confirmed by a partial rescue of oxygen consumption in cells treated with antimycin A (Fig. 3*H*). As stable transfectants of RKO cells no longer expressed RGS4, IL-32 levels were used to assess Cys N-degron pathway regulation in the following experiments. AOX is reported to be more sensitive to O_2_ than cytochrome C oxidase [K_m_O_2_ ~10 to 25 μM (40, 41)]; thus, cells were also exposed to 3% rather than 1% O_2_. AOX expression did not alter IL-32 accumulation under hypoxia alone, but did modestly reduce the effect of NO under hypoxia (Fig. 3*I*). Since AOX functions most efficiently when electron transfer through complex III is blocked (42), we also treated cells with antimycin A. In the absence of AOX, treatment with antimycin A led to reduced IL-32 accumulation under hypoxia and this was not further affected by combined exposure to NO (Fig. 3*I*). However, this effect was attenuated in cells expressing AOX, though IL-32 levels were still slightly lower than that observed in untreated cells (Fig. 3*I*), consistent with the partial restoration of O_2_ consumption. Taken together, these data suggest that NO influences the accumulation of RGS4 by reducing complex IV activity, thereby altering the rate of cytosolic deoxygenation upon exposure to hypoxia.

**Fig. 4.**
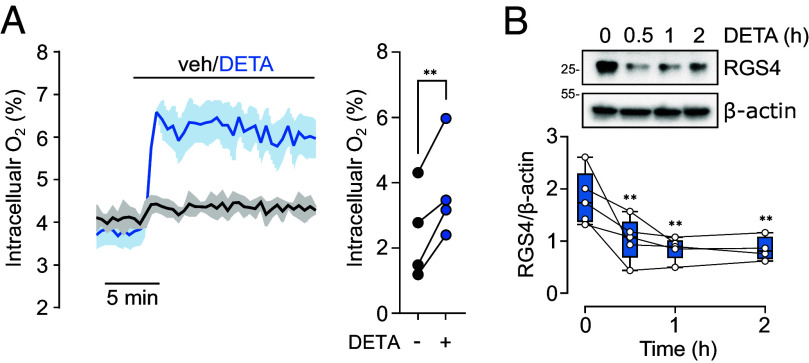
Physiological modulation of oxygenation and RGS4 levels. (*A*) Intracellular O_2_ levels in RKO cells preincubated at 10% ambient O_2_ for 24 h and subsequently treated with vehicle or 100 μM DETA as indicated. A representative trace is provided (shown as the mean ± SD from 3 to 4 replicate wells), and [O_2_]_i_ before and after DETA exposure from four independent experiments shown on the right. **P < 0.01, Paired *t* test. (*B*) RGS4 protein levels in RKO cells exposed to DETA for the indicated periods of time at 10% ambient O_2_ levels. Representative immunoblots are shown above, and the analysis of five independent experiments provided below as the mean ± SD. **P < 0.01, one-way ANOVA with Holm–Šídák’s multiple comparisons post hoc test.

We next considered whether cellular O_2_ availability could be manipulated through alterations in mitochondrial consumption under a constant physiological level of O_2_, and the implications this would have on ADO substrate stability. To this end, we first determined whether acute exposure to NO could induce a meaningful change in [O_2_]_i_ when ambient O_2_ levels were kept at a constant, physiological level (34). While incubation under an atmosphere of 10% O_2_ resulted in substantial variation in measured [O_2_]_i_ in RKO cells (between ~1.5 to 4% O_2_), addition of NO produced a consistent ~50% increase within a short period of time (Fig. 4*A*). As expected based on the relationship between ambient O_2_ levels and ADO substrate accumulation (25), this change in [O_2_]_i_ resulted in a significant decrease in steady-state RGS4 levels in cells cultured under 10% ambient O_2_ (Fig. 4*B*).

## Discussion

The dioxygenation of Nt-Cys controls a proteolytic cascade termed the Cys/Arg N-degron pathway. This reaction was initially described as being both NO and O_2_ dependent (18, 43) and later shown to be catalyzed by PCO in plants (12), and ADO in mammalia (14). These enzymes demonstrate extreme biochemical O_2_ sensitivity, likely explaining the O_2_ dependency of Nt-Cys dioxygenation in cells. However, despite a robust effect of NO on the accumulation of RGS4 during exposure to hypoxia reported previously (14) and here, our data reveal that NO is likely not necessary for Nt-Cys dioxygenation in mammalian cells. This is based on the following observations: First, in vitro dioxygenation of substrate peptides by recombinant ADO can occur in an environment devoid of NO (14, 44). Second, we observed efficient regulation of endogenous substrates in oxygenated cells when NO was efficiently scavenged or prevented from being produced (Fig. 1). Furthermore, addition of NO to cells in which RGS4 was already maximally stabilized (either via severe, prolonged hypoxia or by genetic inactivation of ADO) did not result in its degradation (Figs. 1*C* and 2*E*). Thus, NO alone seems neither necessary nor sufficient to induce Nt-Cys proteolysis in the absence of ADO or O_2_.

Our data suggest that the effects of NO on RGS4/5 protein levels were remarkably dependent on the level of available O_2_, and likely assume a biphasic relationship with cytosolic O_2_ levels as exemplified in Fig. 3*C*. Such a relationship can be inferred from data on RGS4 protein levels, on which a substantial influence of NO was only observed in cells experiencing physiological levels of oxygenation—either in the transition from hyperoxia (atmospheric) to severe hypoxia (1% ambient O_2_, Figs. 1 and 3) or when cultured at a lower, physiological level of O_2_ (Fig. 4). In contrast, conditions in which ambient O_2_ levels were persistently either too high (Fig. 1*A*) or too low (Fig. 2*E*) did not support an action of NO on steady-state RGS4 levels. This complex relationship likely arises from a combination of the i) rapid metabolism of NO in the presence of high levels of O_2_ (2, 3), ii) competitive interaction between NO and O_2_ for binding to cytochrome C oxidase (8), and iii) reduction in mitochondrial respiration as levels approach anoxia (45). Thus, the action of NO to modulate Nt-Cys degron proteolysis is likely restricted to conditions of physiological oxygenation. This work further highlights a prevailing issue in cell culture concerning the disconnect between assumed and actual monolayer oxygenation. Without appropriate consideration, this can lead to the misassignment of a direct effect on cellular oxygen sensing pathways that may be better explained by an indirect impact on O_2_ availability (34, 46, 47).

Several lines of evidence suggest that NO acts at the level of cysteine dioxygenation to affect the stability of substrates of the eukaryotic Cys N-degron pathway. In yeast, which do not possess a PCO- or ADO-like enzyme but otherwise exhibit a fully competent N-degron pathway, NO had no effect on the stability of a Cys N-degron reporter (48). An effect of NO on the Cys/Arg N-degron pathway was originally proposed by Hu et al. (18) using 3T3-NIH fibroblasts. Measurements of pericellular O_2_ levels in cultures of these cells suggest the formation of pronounced O_2_ gradients (49), thus generating conditions permissive for an action of NO on mitochondrial respiration. As these cells have been demonstrated to express NOS and generate NO endogenously (18, 50), inhibition or scavenging thereof would be expected to increase O_2_ consumption, decreasing O_2_ availability further and thus increasing the abundance of ADO substrates. Separately, Jaba et al. (19) described an N-degron-dependent action of both endothelium-derived and exogenously applied NO on RGS4 in cardiomyocytes in vivo and in culture. It has been shown that endothelium-derived NO can modulate oxygen distribution within the surrounding parenchyma through a paracrine action highly sensitive to O_2_ availability (51). In view of this, and the known high O_2_ consumption of cardiac tissue, we believe our data align with observations reported in other mammalian systems.

More work has been conducted on the interactions between NO and the plant Cys N-degron pathway (20, 22, 32, 52). Comparisons between those studies and the current work on mammalian systems are compromised by the considerable physiological differences between eukaryotic systems, as well as the models available to study these mechanisms experimentally. It is also likely that the PCO-ERFVII signaling pathway shares regulatory features common to both ADO and PHD-HIFα pathways operating in mammals (53), with the latter known to be regulated by NO at multiple levels (54).

In summary, we demonstrate that interactions between NO and the mitochondria exert major effects on the availability of O_2_ as an obligate cosubstrate for the Nt-Cys dioxygenase ADO, thus indirectly controlling the stability of its substrate proteins RGS4/5 and IL-32. Our data reveal the importance of this process at physiological oxygen levels in cells, where a dynamic link between mitochondrial respiration and the stability of RGS proteins may represent a regulatory mechanism coupling cellular oxygenation to control of G-protein signaling. This would provide a mechanism for both cell-autonomous homeostatic control, and, in view of the diffusive paracrine properties of NO, also has the potential to contribute to the complex regulation of tissue O_2_ homeostasis. Future studies in intact tissues and organisms should thus be of interest.

### Limitations of the Study.

The biochemistry and physiology of NO signaling are complex and often difficult to comprehensively assess in experimental models. We did not test every possible mechanism through which NO may exert influence over the Cys N-degron pathway, including the possibility of *S-*nitrosothiol modification of ADO or substrate proteins. Moreover, only one source of NO was used to model its actions. We therefore cannot definitively exclude the possibility that NO release through different sources may act upon the Cys N-degron pathway through alternative mechanisms.

## Materials and Methods

### Cell Culture.

RKO, HEK 293T, EA.hy926, and HepG2 cells were cultured in DMEM, and SH-SH5Y cells in DMEM/F12, all supplemented with 10% fetal bovine serum (FBS), 2 mM L-Glutamine, and 100 U mL^−1^ penicillin/10 μg mL^−1^ streptomycin. ADO knock-out cells were generated by CRISPR-Cas9-mediated gene editing as described previously (14). All cell lines were maintained at a 37 °C incubator containing 5% CO_2_, and unless otherwise stated, hypoxic exposure was performed using an atmosphere-regulated workstation set to 1% O_2_: 5% CO_2_: balance N_2_ (Invivo 400, Baker-Ruskinn Technologies).

### Plasmids and Transfections.

A plasmid encoding the mCherry-UBQ-C-RGS4:HA construct was generated by cloning a synthetically produced DNA fragment containing the entire mCherry-UBQ-C-RGS4:HA open reading frame into a pcDNA3+ backbone using restriction digest with *HindIII* and *XbaI* enzymes. The D- and A-RGS4 variants were subsequently produced by cloning a synthetic DNA fragment containing the sequence encoding the UBQ moiety, and the first 60 amino acids of RGS4 (without the initiating methionine) containing the C2D/A mutation, into the above plasmid by *ApoI*-*BamHI* guided restriction digest. For transient expression, 1 μg of either plasmid diluted in 360 μL Opti-MEM and 40 μL PEI (1 mg mL^−1^) was transfected into HEK 293T cells seeded in a 6 cm dish at 70% confluency. 24 h later, cells were split onto a 6-well plate and treated as indicated after an additional 24 h.

RKO cells stably expressing RGS4_1-11_GFP were generated as described previously (25, 35). RKO cells stably expressing doxycycline-inducible, C-terminally FLAG-tagged AOX (AOX:FLAG) were generated through transduction using lentiviral particle-containing supernatant produced by transfecting HEK 293T cells with pCW57-AOX:FLAG (a kind gift from Jessica Spinelli, Program in Molecular Medicine, University of Massachusetts Chan Medical School, Worcester, MA 01605), Addgene plasmid # 177984; http://n2t.net/addgene:177984; RRID:Addgene_177984) alongside the viral packaging plasmids pCMVΔR8.2 and pCMV-VSVG. Transduced cells were selected for by exposure to 0.5 μg mL^−1^ puromycin for 7 d. AOX:FLAG protein expression was subsequently induced by treating cells with 1 μg mL^−1^ doxycycline for 24 h.

### Immunoblotting.

Protein samples were collected in lysis buffer (10 mM Tris pH 7.5, 0.25 M NaCl, 0.5% Igepal) supplemented with Complete™ protease inhibitor cocktail (Sigma Aldrich). Lysates were centrifuged at 13,000 rpm for 3 min at 4 °C, and the supernatant mixed with Laemmli sample buffer. Proteins were then separated via polyacrylamide gel electrophoresis. Membranes were blocked in 4% milk for 1 h, then incubated in primary antibody overnight: HIF-1α (610959, BD Biosciences), RGS4 (15129, CST), IL-32 (sc-517408, SCBT), ADO (ab134102, Abcam), LC3 (L8918, Sigma), FLAG (A8592, Sigma-Aldrich), and HA (3F10, Roche). horseradish peroxidase-conjugated secondary antibodies were sourced from DAKO and used in conjunction with chemiluminescence substrate (West Dura, 34076, Thermo Fisher Scientific) to visualize protein expression using a ChemiDoc XRS+ imaging system (BioRad). β-actin primary antibody was conjugated directly to horseradish peroxidase (ab49900, Abcam). Native mCherry fluorescence was assessed in-gel by monitoring emitted light at 605 ± 50 nm prior to transfer. Densitometric analysis was performed using ImageJ software (NIH) and values presented relative to β-actin.

### RT-qPCR.

Tri-Reagent (T9424, Sigma Aldrich) was used to extract RNA by phase separation, and complementary DNA (cDNA) synthesis was performed using equal quantities of RNA using the High-Capacity cDNA Kit (Applied Biosystems). qPCR analysis was performed using Fast SYBR Green Master Mix on a StepOne thermocycler (Thermo Fisher Scientific) using the ΔCt method. The housekeeping gene Hypoxanthine-guanine phosphoribosyl transferase (HPRT) was used as a reference. Sequences for the primers used are as follows:

RGS4 (F_GCAAAGGGCTTGCAGGTCT, R_CAGCAGGAAACCTAGCCGAT),

NOS1 (F_ATTTATGCCGCGTTTCCAGC, R_AGGCATCATGAGCCCGTC),

NOS2 (F_CGTGGAGACGGGAAAGAAGT, R_CCTGGGTCCTCTGGTCAAAC),

NOS3 (F_GACCCACTGGTGTCCTCTTG, R_CCCGAACACACAGAACCTGA),

HPRT (F_GACCAGTCAACAGGGGACAT, R_AACACTTCGTGGGGTCCTTTTC*),*

AOX1 (F_GGGGTGTTCCGTGTTTTTCG, R_CAGGTTCATCTCTCGGAATCATTT),

CYGB (F_ACCGGTGTACTTCAAGATCCTC, R_ACCCAGAAATGGAGCGTCAA),

MB (F_CCAGTGAGCCCATACTTGCT, R_GGTGACCCTTAAAGAGCCTGAT),

MTARC1 (F_TTTGTCCTCCTCGCGCAAT, R_CACAAGCCAAAACCTGTCCC),

MOCOS (F_TTCCTGGTACAATGGCCACC, R_AAGGCCCCAAAACCTGGAAA),

MOCS2 (F_TTCGGTCCCGCTGTCCTA, R_TCTAACATCAGCCAATCCAGG),

MOCS3 (F_CAGCCACTGATAAATGCCGC, R_CAGGTGGAATGCCCCAGAAT),

NGB (F_CCTGTTTGCCAGGCTGTTTG, R_CTGCAGCATCAATCACGAGC),

SUOX (F_CACTGAGTTCACCTGCGAGT, R_AGGGGATTGACTTGAGTCTGC),

XDH (F_AGCACTAACACTGTGCCCAA, R_TGGTCTGACAAGCCGCATAG)

### Measurement of Intracellular Oxygen Levels.

RKO cells were seeded in triplicate or quadruplicate wells of black, clear-bottomed 96-well plates and allowed to reach 80% confluency. Cells were loaded with MitoXpress INTRA (MX-300-4, Agilent) at a final concentration of 10 μg mL^−1^ diluted in standard DMEM +10% FBS for 16 h, after which time any probe not taken up was washed off with phosphate buffered saline. Cells were then maintained in low autofluorescence FluoroBrite DMEM (A1896701, Gibco) with 1% FBS and containing either DMSO vehicle (0.1%), 100 μM DETA (82120, Cayman Chemicals), or 1 μM antimycin A (A8674, Sigma) and placed in either a CLARIOstar (BMG Labtech) or Spark (Tecan) plate reader set at 37 °C and 5% CO_2_. MitoXpress INTRA was excited at 365 nm and the phosphorescence emitted at 650 nm was measured within 30 μs windows at two delay times (30 μs/t_1_ and 70 μs/t_2_). Raw phosphorescence was converted to lifetime using the following formula: Lifetime = (t_2_ – t_1_)/ln(f_1_/f_2_), where f_1/2_ represents the measured intensity at t_1/2_. Lifetime was subsequently converted to %O_2_ using the formula: %O_2_ = 659.3*Exp(−lifetime/8.475), as described by the manufacturer. In experiments shown in Fig. 3*B*, baseline intracellular O_2_ levels were assessed at atmospheric levels of O_2_ in standard medium, or medium containing either 1 μM antimycin A, 100 μM DETA, 200 μM cPTIO (81540, Cayman Chemicals), or DETA+cPTIO, before purging the internal plate reader atmosphere to 1% O_2_ and allowing intracellular O_2_ levels to equilibrate over a further hour. In experiments shown in Fig. 3*C*, cells were treated with 100 μM DETA or control (medium alone) and subjected to step-wise reductions in the ambient O_2_ level from 18.5 to 1% O_2_ over a ~5 h period. At each O_2_ level, [O_2_]_i_ was allowed to plateau before any further reduction in ambient O_2_ level was performed. In experiments shown in Fig. 3*H*, cells were induced with doxycycline (1 μg mL^−1^) for 24 h prior to loading with MitoXpress INTRA, then treated with 1 μM antimycin A. Intracellular O_2_ was monitored for 1 h to obtain an average. In experiments shown in Fig. 4*A*, cells were pre-equilibrated for 24 h in a hypoxia workstation set to 10% O_2_ before being rapidly transferred to the plate reader also set to 10% O_2_. Intracellular O_2_ levels were allowed to settle for 1 h, before DETA was injected to a final concentration of 100 μM (15 μL of 1 mM stock injected into 135μL medium). 10 μM NaOH in FluoroBrite medium was used as a vehicle control.

### ADO Substrate Reporter Assay.

RKO cells stably expressing RGS4_1-11_GFP (25, 35) were seeded in triplicate in black, clear-bottomed 96-well plates and allowed to reach 80% confluency. For experiments investigating the effect of medium volume on reporter accumulation, culture medium was replaced with the indicated volume of FluoroBrite DMEM +1% FBS alone, or supplemented with either 100 μM 2,2-dypyridyl or 100 μM DETA. Cells were then subjected to 4 h at 1% O_2_ before fluorescence was measured at 480 nm excitation, 520 nm emission using a FLUOstar Omega plate reader (BMG Labtech). Real-time measurements of reporter levels were performed as above, but using a CLARIOstar plate reader (BMG Labtech) with atmospheric control, set to 5% CO_2_ at 37 °C. After 1 h, the atmosphere was switched to 1% O_2_: 5% CO_2_ and GFP fluorescence monitored every 5 min for 16 h.

## Data Availability

All study data are included in the main text.
